# Health consequences of female genital mutilation/cutting in the Gambia, evidence into action

**DOI:** 10.1186/1742-4755-8-26

**Published:** 2011-10-03

**Authors:** Adriana Kaplan, Suiberto Hechavarría, Miguel Martín, Isabelle Bonhoure

**Affiliations:** 1 Cátedra de Transferencia del Conocimiento/Parc de Recerca UAB-Santander, Departamento de Antropología Social y Cultural, Universitat Autònoma de Barcelona, Barcelona, Spain; 2 Grupo Interdisciplinar para la Prevención y el Estudio de las Prácticas Tradicionales Perjudiciales (GIPE/PTP), Departamento de Antropología Social y Cultural, Facultad de Letras y Psicología, Universitat Autònoma de Barcelona, Barcelona, Spain; 3 NGO Wassu Gambia Kafo, Fajara F Section, Banjul, The Gambia; 4 Cuban Medical Mission in The Gambia, Banjul, The Gambia; 5 Community Based Medical Program, Ministry of Health and Social Welfare, Banjul, The Gambia; 6 Facultad de Ciencias Médicas Manuel Fajardo. Universidad Médica de la Habana, La Habana, Cuba; 7 Grups de Recerca d'Amèrica i África Llatines (GRAAL), Unitat de Bioestadistíca. Facultat de Medicina, Universitat Autònoma de Barcelona, Barcelona, Spain

**Keywords:** Female Genital Mutilation/Cutting, Gambia, Sexual and Reproductive Health, Africa

## Abstract

**Background:**

Female Genital Mutilation/Cutting (FGM/C) is a harmful traditional practice with severe health complications, deeply rooted in many Sub-Saharan African countries. In The Gambia, the prevalence of FGM/C is 78.3% in women aged between 15 and 49 years. The objective of this study is to perform a first evaluation of the magnitude of the health consequences of FGM/C in The Gambia.

**Methods:**

Data were collected on types of FGM/C and health consequences of each type of FGM/C from 871 female patients who consulted for any problem requiring a medical gynaecologic examination and who had undergone FGM/C in The Gambia.

**Results:**

The prevalence of patients with different types of FGM/C were: type I, 66.2%; type II, 26.3%; and type III, 7.5%. Complications due to FGM/C were found in 299 of the 871 patients (34.3%). Even type I, the form of FGM/C of least anatomical extent, presented complications in 1 of 5 girls and women examined.

**Conclusion:**

This study shows that FGM/C is still practiced in all the six regions of The Gambia, the most common form being type I, followed by type II. All forms of FGM/C, including type I, produce significantly high percentages of complications, especially infections.

## Background

"Female Genital Mutilation/Cutting" (FGM/C) refers to all procedures involving partial or total removal of the external female genitalia or other injury to the female genital organs for non-medical reasons [1]. It is recognized internationally as a violation of the human rights of girls and women and constitutes an extreme form of discrimination against women due to the severe health consequences and the pain and risks involved.

WHO calculates that between 100-140 million women and girls in the world have been victims of some kind of FGM/C and that each year about 3 million girls are at risk or are subjected to some kind of FGM/C, essentially in 28 countries in sub-Saharan Africa, northern Iraq (Kurdistan), Malaysia and Indonesia, plus Europe, USA and Australia among many other countries where migrants carry along their culture [2,3]. In many societies it is a rite of passage to womanhood with strong, ancestral sociocultural roots. Rationalizations for the perpetuation of FGM/C include: preservation of ethnic and gender identity, femininity, female purity/virginity and "family honour"; maintenance of cleanliness and health; and assurance of women's marriageability [4,5]. In the Gambia, FGM/C is carried out in girls aged between birth (7 days) up to pre-adolescence, always before the first menstruation and marriage [6-9].

According to the WHO classification of 1995 [10] used in this study designed in November 2008, FGM/C can be divided into four types:

Type I: Excision of the prepuce and part or all of the clitoris.

Type II: Excision of the prepuce and clitoris together with partial or total excision of the labia minora.

Type III: Infibulation. Excision of part or all of the external genitalia and stitching together of the two cut sides, to varying degrees.

Type IV: Pricking, piercing, incision, stretching, scraping, or other procedures harming the clitoris or labia, or both.

Types I, II and III of FGM/C present severe health consequences that have been well documented by several authors. The immediate health complications include shock, haemorrhage, infections and psychological consequences [11-13]. The long term health risks consist of chronic pain, infections, cheloids formation, primary infertility, birth complications, danger to the new born and psychological consequences [13-18]. Even FGM/C types I and II, sometimes considered as more innocuous, may involve severe health complications. For example, they have been reported to provoke unequivocal complications like shock, haemorrhage, urogenital complications [12], obstetric complications [18] and sexual dysfunction [15].

Despite the documented adverse effects of FGM/C, a recent review article states that its overall prevalence has declined very little [19].

In the case of The Gambia, the survey MICS 2006 shows that the prevalence of FGM/C remains as high as 78.3% in women aged between 15 and 49 years [20]. In 1999, Morison et al. conducted a community-based survey on the long-term health consequences of FGM/C in rural Gambia [16]. The conclusions were that women who had undergone FGM/C had higher prevalence of bacterial vaginosis and of herpes simplex virus 2 (HSV2). Nevertheless, knowledge about the extent of health consequences of FGM/C in The Gambia is scarce.

This issue has political and religious implications since the government of The Gambia asked for medical proofs on the harmfulness of the practice in order to permit making an informed statement based on local evidence and taking further steps. The request for this study to be conducted was made by the Vice-President and Minister of Women's Affairs, Dr. Aja Isatou Njie-Saidy. The clinical evidence will lend support to the political will to prevent the practice of FGM/C in The Gambia through education and legislation.

Thus, the main purpose of the present study is to analyze the health consequences of FGM/C in patients who spontaneously sought medical care. The specific objectives are to: 1) find out if the practice still exists in the country 2) determine which types of FGM/C are practiced 3) determine what health problems are associated to each type.

## Methods

### Design of the study

Previous studies evidenced the imprecision of self-reported FGM/C and its health consequences [21]. Thus, the study has been designed to permit ascertaining FGM/C types and health consequences directly, through a medical examination. The data has been collected during 4 months (December 2008 to March 2009) in hospitals and major health centres throughout the country, achieving a total of 871 cases of women and girls that have undergone the practice.

### Population

women and girls who sought consultation for whatever problem requiring a medical gynaecological examination and who had undergone FGM/C. As there is no national health register, it was impossible to determine the total number of medical consultations during study period, consequently making it impossible to calculate morbidity and mortality indices.

### Data collection

The data has been collected by Cuban doctors, specialized in General Comprehensive Medicine and Gynaecologists, working in The Gambia on a humanitarian mission. These doctors were previously trained in order to be able to clearly identify the different types of FGM/C performed.

The data analyzed here are obtained from secondary data as a result of patients' spontaneous demand and consequently the use of the data no required previous information to the patient. Furthermore, the research team, in agreement with the Gambian health authorities, collected data anonymously.

After the doctors visited the patients, the information was collected in a clinical form specially designed for the study. They could not guarantee that the data was recorded exhaustively due to the inadequate conditions of the health facilities and the over demand of medical services. This survey provides a "raw" picture and is the preliminary step to conducting a more complete clinical survey, covering complications during delivery and foetal suffering.

### Clinical examination

The types and complications of FGM/C were assessed by direct clinical exam of the genitalia. The review took place only in medical facilities (consultations and delivery rooms). For every patient with FGM/C that had haemorrhage and or anaemia as their chief complaint, a FBC (Full Blood Count) was done to confirm if they were anaemic or not. The infections were diagnosed clinically, and any long term complications expressed by the patients were also recorded, although none of them associated their complaint to the practice they undergone when they were young, except for girls who came immediately after FGM/C had been performed. The clinical examination only was done if the patient reported a medical problem requiring a medical gynaecological examination.

### Variables studied

#### 1. Demographic Variables

- Registration place: Patient's region of residence. All six regions in The Gambia: Upper River, Central River, Low River, North Bank, Western and Greater Banjul.

- Ethnic group: Mandinga, Wolof, Fula, Djola, Saraholes, Serer and Others.

#### 2. Clinical variables (qualitative variables)

- Type of cutting (ordinal). Categories of this variable were those defined by WHO in 1995. Type IV was not included in the study.

- Complications. Complications in patients with cuttings, dichotomized variable (yes/no). Complications were also classified, according to the time when they appeared, into:

*• Immediate*. Patients in who cutting was recent, with signs of complication appearing in the next few hours and up to 10 days after cutting was performed.

*• Long Term*. Complications appeared more than 10 days later, and were more related with pregnancy affectations during labour or childbirth (Obstetrics complications).

- Type of complications. Depending on aetiology, the complications can be:

*• Haemorrhage*. Excessive bleeding from genitalia because of this cause.

*• Acute Anaemia*. Considered in this study if the patient presented symptomatology suggesting anaemia, as a consequence of haemorrhage due to the cutting. Based on the results of the differential FBC, a patient was considered anaemic if their haemoglobin level was below 11 g/l, and non-anaemic otherwise.

*• Infections*. Invasion and spreading of pathogenic micro organisms, divided into:

- Tetanus: Presence of fits or convulsions, opistotonous position.

- Repetitive infections of low urinary tract.

- Infection of the urethral mucus and/or cystitis.

- Septicaemia: Colonization of the blood by bacteria with a lethal systemic infectious stage.

- Vulvovaginitis: Acute inflammation of the vaginal mucus characterized by burnings, itching, redness and excoriation due to rash with or without leucorrhoea.

• Abnormal scarring:

- Fibrosis: Pathologic formation of fibrous tissue in the genitalia due to an abnormal scarring or cicatrisation of cuts, i.e. limited to the site of cutting.

- Cheloids. Excessive growth of scar tissue at the site of a skin lesion that has just healed. Characterized by an abnormal growth of the tissue over the place of the cutting.

- Synechia: Adherence and abnormal fusion which may be partial or total, of the major or minor labia.

- Tissue rotation: Due to the tissue lost with abnormal scarring and retraction of anatomical zones.

• Organic dispareunia: Gynaecological complication characterized by pain during intercourse due to the cutting.

### Statistical treatment of the data

The data have been analysed in aggregated crosstabs classifications looking for the major associations between type of FGM/C and possible health injuries as listed above. Chi square tests of association were calculated and partial binomial and multinomial distributions are compared.

### Ethical aspects

This survey was done at the request of the Gambian government. The clinical register was kept under the custody of the medical personnel in charge of that issue, and under rigorous confidentiality.

## Results

Data was collected on a total of 871 cases, throughout the country. The types of FGM/C and health complications deriving from them are summarized in Table 1. Type I FGM/C accounted for 66.2% of the cases registered, and type II 26.3%. Type III had a much lower prevalence, 7.5%. A substantial number of cases were observed with health complications arising directly from the practice of FGM/C. Complications, whether immediate or late, were present in 23.7% of the patients with type I FGM/C (137/577), in 55.0% of patients with type II (126/229) and in 55.4% of patients with type III (36/65). The most common immediate complication, for all types, was infection, associated in some cases with haemorrhage and anaemia. 110 patients out of the 871 registered patients, i.e. 12.6%, sought consultation for an immediate complication of FGM/C. The late complications were observed in 189 patients out of 871 patients, 21.7%. In total, 299 out of 871 registered patients with FGM/C who sought a gynaecological consultation (34.3%), presented immediate or late complications due to FGM/C.

**Table 1:** Number of cases by FGM/C types, complications (total, immediate and late) and types of complications

	FGM/C type			TOTAL
	**I**	**II**	**III**	

**Cases**	577 (66.2%)	229 (26.3%)	65 (7.5%)	871 (100%)

**Total complications directly arising from FGM/C**^**a**^	**137** **(23.7%)**	**126 (55.0%)**	**36 (55.4%)**	**299 (34.3%)**

**Immediate complications**^**b**^	**36** **(26.3%)**	**55 (43.7%)**	**19 (52.8%)**	**110 (36.8%)**

Infections^c^	32 (88.9%)	48 (87.3%)	16 (84.2%)	96 (87.3%)

Haemorrhage^c^	10 (27.8%)	23 (41.8%)	7 (36.8%)	40 (36.4%)

Anaemia^c^	15 (41.7%)	17 (30.9%)	10 (52.6%)	42 (38.2%)

**Late complications**^**b**^	**101** **(73.7%)**	**71 (56.3%)**	**17 (47.2%)**	**189 (63.2%)**

Abnormal scarring^d^	87 (86.1%)	63 (88.7%)	11 (64.7%)	161 (85.2%)

a.- of the total cases, b.- of the total complications (immediate and late) c.- of the total immediate complications, d.- of the total late complications.

## Discussion

These findings, and in particular the immediate complications presented by young girls, demonstrates that FGM/C is still practiced in all the six regions of The Gambia.

The clinical examinations evidenced that, out of the women and girls who presented signs of FGM/C, the majority had FGM/C type I (66.2%), followed by type II (26.3%) and type III (7.5%). The results present discrepancies with Morison's survey, in which 98% of the women that presented FGM/C had type II [16]. As that study was performed in a specific area, Farafenni (Northbank East), direct comparison with the present work, covering the whole country, is not possible. However, the percentages of type I and II are comparable with others studies performed in West Africa, namely Ghana and Nigeria [22,23] and with the fact that FGM/C type III is only the most prevalent form in East Africa.

Another finding of this survey is that all the forms of FGM/C, including type I, are responsible for a high percentage of complications, both immediate and late. In this study, 110 immediate complications and 189 late complications, out of 871 cases, were observed. In particular, immediate FGM/C complications, reported previously [11,12], appear to be significant in this study. It has to be noted that even type I FGM/C, the form of FGM/C with least anatomical extent, presented complications in 1 of every 5 girls and women who consulted. Complications in type II FGM/C were observed in 1 of every 2 girls and women examined, thus confirming the previous finding that the prevalence of health consequences are proportional to the anatomical extent of FGM/C [1]. For type III, which was only present in a total of 65 patients, complications are also observed in 1 of every 2 girls and women. However, because of the size of the sample for type III, this figure could be 2 of every 3, i.e. corresponding to the upper limit of CI_95%_. It has to be stressed that these figures are purely indicative as the patients are not necessarily representative of all girls and women who have undergone the practice of FGM/C. Nevertheless, these results give a first hint of the frequency of health consequences arising from any form of FGM/C in The Gambia. Moreover, the results that we report only include girls and women who had access to a medical facility, something which is difficult for most of the rural population. In The Gambia, the majority of the population live in rural areas, often badly communicated and far from any dispensary, where the prevalence of FGM/C has been documented to be higher, and in the eastern regions, almost universal [20]. For this rural population, it can be hypothesized that the health consequences related to FGM/C are more severe due to the lack of adequate medical care "post-intervention" as previously stated by other authors [14] and as it was demonstrated for others reproductive organ pathologies [24] and for maternal mortality in rural Gambia [25,26].

This study also indicates that the practice of FGM/C has a significant economic cost as 1 of 3 patients (299 cases of 871) suffered medical consequences requiring treatment. This finding was corroborated by a recent study showing that the annual costs of FGM/C-related obstetric complications ranged from 0.1 to 1% of government spending on health for women aged 15-45 years. Moreover, in the six African countries where the study was done, e.g. Burkina Faso, Ghana, Kenya, Nigeria, Senegal and Sudan, a loss of 130,000 life years is expected owing to FGM/C's association with obstetric haemorrhage [27].

Given such adverse effects, one is tempted to ask why the practice of FGM/C is still continuing. Certainly, one of the reasons would be that FGM/C has strong ancestral socio-cultural roots as evidenced by the fact that 72.9% of the Gambian women would like their daughters to undergo FGM/C [20]. Another reason is probably the lack of knowledge regarding health consequences associated with FGM/C, even among local health professionals like nurses or midwifes regarded by a recent review as a specific target to be addressed in order to favour abandonment of the practice [19]. These preliminary results will allow the implementation of a national training work plan for the health professionals and students regarding the issue of FGM/C, directly based on the observed health consequences in The Gambia.

## Conclusions

FGM/C is still practiced in all the six regions of the country and resulted in various forms of damage/injury in 1 of every 3 of the women examined.

The form of FGM/C most commonly practiced in The Gambia is type I, followed by type II. All forms of FGM/C, even type I, lead to a high percentage of complications, especially the infections associated with haemorrhage and anaemia. The frequency of complications increases with the degree of mutilation/cutting.

## Competing interests

The authors declare that they have no competing interests.

## Authors' contributions

AK performed the previous studies about FGM/C, proposed and designed the clinical study on health consequences of FGM/C in The Gambia, performed the training of the medical doctors in the field and participated to the data treatment and analysis.

SH participated to the design of the clinical study, organized the implementation of the data collection in the field and participated to the data treatment and analysis.

MM analyzed the data, in collaboration with the other authors and reviewed the manuscript.

IB wrote the first draft of the article and coordinated the review and publication process.

All authors read and approved the final manuscript.
